# A new class of small molecule estrogen receptor-alpha antagonists that overcome anti-estrogen resistance

**DOI:** 10.18632/oncotarget.6323

**Published:** 2015-11-13

**Authors:** Yongxian Ma, Anju Preet, York Tomita, Eliseu De Oliveira, Li Zhang, Yumi Ueda, Robert Clarke, Milton Brown, Eliot M. Rosen

**Affiliations:** ^1^ Department of Oncology, Georgetown University School of Medicine, Washington, DC, USA; ^2^ Department of Biochemistry and Molecular and Cellular Biology, Georgetown University School of Medicine, Washington, DC, USA; ^3^ Department of Radiation Medicine, Georgetown University School of Medicine, Washington, DC, USA; ^4^ Department of Center for Drug Discovery Georgetown University School of Medicine, Washington, DC, USA

**Keywords:** BRCA1, estrogen receptor (ER-α), antagonist, agonist, Tamoxifen

## Abstract

Previous studies indicate that BRCA1 protein binds to estrogen receptor-alpha (ER) and inhibits its activity. Here, we found that BRCA1 over-expression not only inhibits ER activity in anti-estrogen-resistant LCC9 cells but also partially restores their sensitivity to Tamoxifen. To simulate the mechanism of BRCA1 inhibition of ER in the setting of Tamoxifen resistance, we created a three-dimensional model of a BRCA1-binding cavity within the ER/Tamoxifen complex; and we screened a pharmacophore database to identify small molecules that could fit into this cavity. Among the top 40 “hits”, six exhibited potent ER inhibitory activity in anti-estrogen-sensitive MCF-7 cells and four of the six exhibited similar activity (IC_50_ ≤ 1.0 μM) in LCC9 cells. We validated the model by mutation analysis. Two representative compounds (4631-P/1 and 35466-L/1) inhibited ER-dependent cell proliferation in Tamoxifen-resistant cells (LCC9 and LCC2) and partially restored sensitivity to Tamoxifen. The compounds also disrupted the association of BRCA1 with ER. In electrophoretic mobility shift assays, the compounds caused dissociation of ER from a model estrogen response element. Finally, a modified form of compound 35446 (hydrochloride salt) inhibited growth of LCC9 tumor xenografts at non-toxic concentrations. These results identify a novel group of small molecules that can overcome Tamoxifen resistance.

## INTRODUCTION

At presentation, about 70% of breast cancers are estrogen receptor-positive (ER+) and thus suitable for anti-estrogen therapy [1]. Despite being ER+, 50% of patients with advanced breast cancer who receive anti-estrogen treatment with Tamoxifen (Tam) fail to respond; and all patients with metastatic breast cancer eventually develop Tam resistance. In addition, many patients (about 40%) who receive Tam as adjuvant therapy will relapse and die of disease [1]. The causes of resistance to different anti-estrogens are not identical, but cross-resistance is common [1]. In most cases of acquired anti-estrogen resistance, breast cancers retain ER and may be amenable to novel approaches to target ER.

Mutations of *BRCA1* account for half of all hereditary breast cancers [2]; and in 30-40% of sporadic cancers, BRCA1 expression is absent or reduced, suggesting a wider role in breast cancer [3-6]. While many studies on BRCA1 have focused on its roles in maintenance of genomic integrity [7, 8], BRCA1 also functions to regulate ER activity. Thus, a mammary-targeted *Brca1* deficiency confers hypersensitivity to estrogen and promotes the development of mammary pre-neoplasia and cancer in mice [9]. In cultured cells, BRCA1-siRNA causes estrogen-independent ER activation and stimulates the agonist activity of Tam; and in *Brca1*-deficient mice, Tam promotes mammary cancer development [10, 11]. Finally, BRCA1-knockdown causes Tam-resistance due to altered recruitment of co-regulators by ER [12].

Based on studies of the BRCA1: ER interaction, we identified small molecules that recapitulate the mode in which a portion of BRCA1 inserts into ER and inhibits its activity. We identified two distinct non-overlapping sets of compounds, one based on ER ligated to 17β-estradiol (E2) [13] and the other based on ER bound to 4OH-Tam. This manuscript focuses on the latter compounds that bind to the ER/Tam complex. These compounds do not bind to the ligand-binding pocket and thus work differently from conventional anti-estrogens such as Tam and Fulvestrant. Here we report on initial characterization of the activity of these compounds.

## RESULTS

### BRCA1 over-expression partially restores Tam sensitivity to anti-estrogen-resistant LCC9 cells

**Figure 1:**
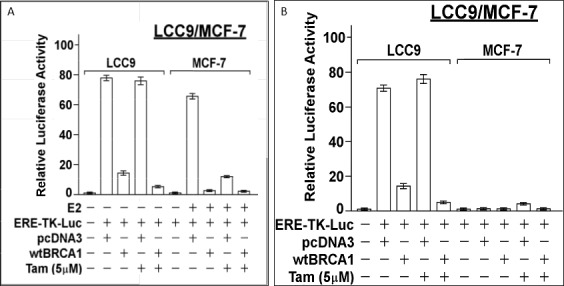
Inhibition of ER activity in LCC9 and MCF-7 cells by BRCA1 LCC9 or MCF-7 cells in 24-well dishes were co-transfected overnight with the ERE-TK-Luc reporter plasmid and wild-type (wt) BRCA1 or empty pcDNA3 vector (0.25 μg of each plasmid per well), washed, and allowed to recover for several hours in fresh culture medium (DMEM plus 5% charcoal-stripped serum). The cells were then treated ± 17β-estradiol (E2, 10 nM) and ± Tamoxifen (5 μM), as indicated for 24-hr, after which the cells were harvested for luciferase assays. For MCF-7, luciferase activity is expressed as a fold-change relative to the no E2 control. For LCC9, luciferase activity is expressed relative to the control without the reporter present. Values plotted are means ± SEMs of four replicate wells. The data shown in each panel are representative of three independent experiments.

We compared the effect of BRCA1 over-expression (by transfection of wild-type (wt) BRCA1 vector) in LCC9 human breast cancer cells with anti-estrogen sensitive MCF-7 cells. wtBRCA1 suppressed the constitutive ER activity (measured using ERE-TK-Luc reporter), but Tam (5 μM) alone had no effect (Figure 1A and 1B (*left*)). The combination of wtBRCA1+Tam gave greater suppression of ER activity than wtBRCA1 alone (*P* < 0.001). In contrast to LCC9, wtBRCA1 and Tam each strongly suppressed E2-stimulated ER activity in MCF-7 cells. When MCF-7 cells were tested in the absence of E2, ER activity was very low under most conditions (illustrating the requirement for E2 to activate ER); but without E2, Tam functioned as an ER agonist and caused a (5-6)-fold increase in ER activity (*P* < 0.001). Thus, BRCA1 inhibits ER activity in LCC9 cells and partially restores sensitivity to Tam.

### New set of BRCA1-related ER antagonists

An original set of compounds were designed to mimic a portion of BRCA1 in complex with E2-bound ER [13]. We reasoned that since the conformation of ER bound to Tam differs from that of E2-bound ER [14], a screening of compounds based on the Tam-bound ER might identify compounds whose binding to ER would synergize with Tam and help re-sensitize resistant breast cancers to Tam. We expected that the chemical structures of new compounds that bind to the BRCA1 cavity on the Tam-bound ER would differ from the original compounds as the shape and characteristics of the putative BRCA1-binding cavities are distinct.

### *In silico* screening of small molecules

Based on the model structure of the BRCA1-binding interface of ER ligand-binding domain (LBD) in complex with 4OHTam, we set up an *in silico* screening of small molecule libraries. Based on our successful previous screening [13], we defined the small drug-like molecule binding site that is close to the BRCA1-binding interface and the E2-binding pocket. This site is essentially the same location on ER as the previously described site, but it is altered due to the OHTam binding to ER. Of note is the relative location of these two sites, which form two separate pockets, but because of their physical proximity and the fact that the BRCA1 pocket site is defined in the presence of 4OHTam, the binding of a small molecule at the BRCA1 site and that of 4OHTam are expected to synergistically influence their binding properties and mimic BRCA1 suppression of ER. We conducted a virtual screening against the BRCA1 pocket site using the National Cancer Institute/Developmental Therapeutics Program “Diversity Set”. This screening yielded the 40 top ranked compounds (selection criteria are described in the Methods section), Even though we screened the same database of 1,990 compounds in the same manner as before, there was no overlap between the new set of the top 40 compounds identified based on the ER/4OHTam structure and the original set of the top 40 compounds based on the ER/E2 structure.

### Screening of compounds for inhibition of ER activity

**Figure 2:**
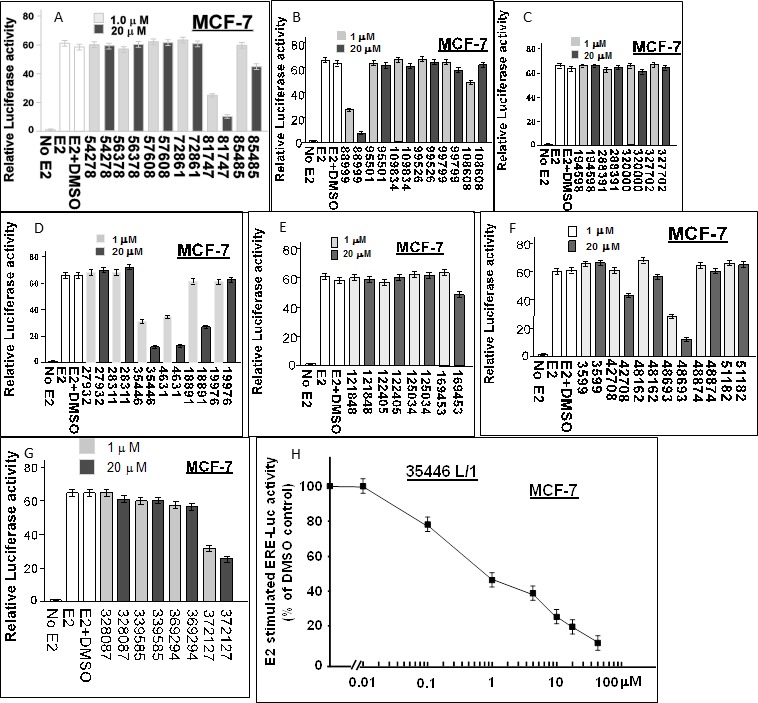
Screening of 36 candidate compounds (“hits”) for inhibition of E2-stimulated ER-α activity in MCF-7 human breast cancer cells MCF-7 cells in 24-well dishes were transfected overnight with the ERE-TK-Luc reporter plasmid (0.25 μg per well), washed, and allowed to recover for several hours in fresh culture medium. The cells were then treated ± 17β-estradiol (E2, 10 nM) and with the indicated compound (@ 1 μM or 20 μM) or vehicle only (DMSO) for 24 hr and assayed for luciferase activity. Luciferase activity is expressed as a fold-change relative to the no E2 control. The data shown are representative of three independent experiments.

**Figure 3:**
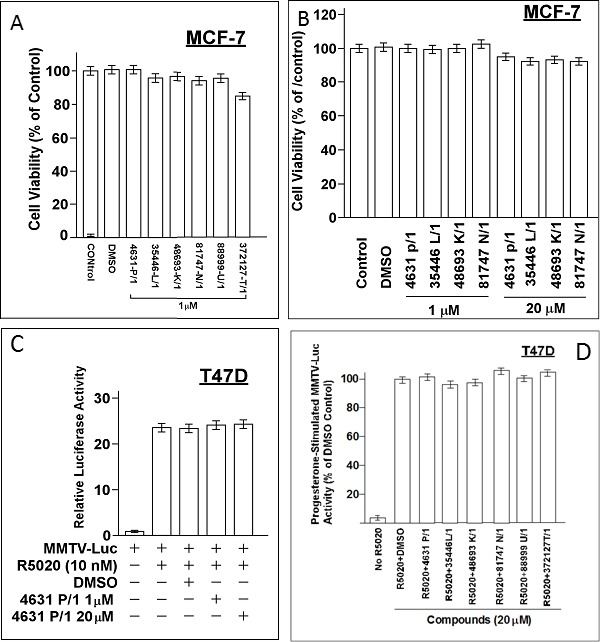
Cytotoxicity and specificity of compounds **A.**, **B.** Subconfluent proliferating cells were incubated with the indicated compound at the indicated concentration for 24-hr and then harvested for MTT dye reduction assays. Cell viability values (relative to untreated control cells) are means ± SEMs of 10 replicate wells. **C.**, **D.** Subconfuent proliferating T47D cells were transfected overnight with the MMTV-Luc reporter (0.25 μg per well in 24-well dishes), washed, and allowed to recover for several hr in fresh medium. The cells were then treated with a synthetic progestin (R5020, 10 nM) plus the indicated compound at the indicated concentration for 24 hr and then harvested for luciferase assays. Luciferase activity is expressed relative to the MMTV-Luc control (no agents added) (panel A) or as a percentage of the +R5020 positive control (panel B). Values are means ± SEMs of four replicate wells. The data shown in each panel are representative of three independent experiments.

We obtained 36 of the top 40 “hit” compounds from the NCI and tested them for inhibition of E2-stimulated ER activity in MCF-7 cells. Six of the 36 compounds (4631-P/1, 35466-L/1, 48693-K/1, 81747-N/1, 88999-U/1, and 372127-T/1) gave ≥ 50% inhibition at 1 μM, indicating IC_50_ values ≤ 1 μM (Figure 2). All six compounds gave > 80% inhibition at 20 μM. The remaining 30 compounds had no inhibitory activity or had IC_50_ values ≥ 20 μM. A complete dose-response curve is shown for one compound (35466-L/1) (Figure 2H). This curve shows a continuous reduction of ER activity as the concentration of compound 35446-L/1 is increased from 0.01 to 50 μM, with an IC_50_ of 0.8 μM. It is unlikely that the ER inhibition is due to non-specific cytotoxicity, since cell viability ranged from 85-100% after a 24-hr exposure to 1 μM of each of the compounds; and for four of the six compounds (4631-P/1, 35446-L/1, 48693-K/1, and 81747-K/1), cell viability was 100% at a 1 μM concentration and over 90% at 20 μM (Figure 3A-3B). We tested each of the six active compounds for its ability to inhibit progestin-stimulated progesterone receptor (PR) activity in a PR+ breast cancer cell line (T47D), using the MMTV-Luc reporter. There was little or no inhibition of PR activity by any of the compounds (Figure 3C-3D), suggesting that ER inhibition is selective.

### Structural characteristics of interaction of compounds with ER

**Figure 4:**
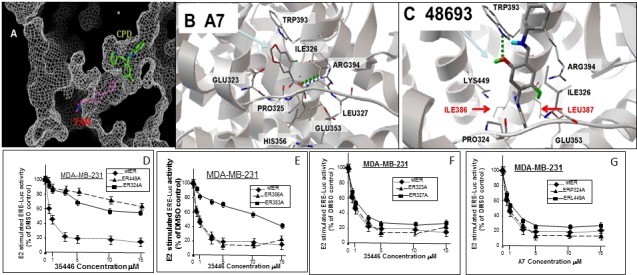
Interaction of new compounds with ER **A.** A cut-through surface model of ER (in gray) showing the distinct locations where our compounds (CPD, in green) as opposed to Tamoxifen (TAM, in magenta) bind to ER. **B.**, **C.** Panels B and C show a comparison of the ER interaction of one of our original series of compounds (A7) (B) and one new compounds (48693-K/1) (C). ER is represented by gray ribbon and its residues interacting with the compounds are drawn with wireframe and labeled. Panel B shows the ER structure based on E2 bound form (PDB code: 1ERE), which is docked with A7. The hydrogen bond interaction with ARG 394 is shown in green line. Panel C shows the ER structure based on Tam bound form (PDB code: 3ERT), which is docked with 48693-K1. Two unique residues interacting with ER are shown with red label (these are commonly used in ER interactions with all of the new compounds docked to ER/Tam (C) but not with the original compounds docked to ER/E2 (B). The hydrogen bond interaction of compound 48693-K/1 with TRP393 is also shown in green line. **D.**-**G.** ER-negative cell line MDA-MB-31 was transfected overnight with wild-type ER (wtER) or the indicated point mutant ER plus the ERE-TK-Luc reporter. Cells were treated with E2 (10 nM) and different concentrations of compound 35466-L/1 for 24-hr in phenol red-free DMEM with 5% charcoal-stripped serum (CSS). The cells were harvested for luciferase assays. Luciferase values are expressed as a percent of the +E2 vehicle control and are means ± SEMs of four wells. The data shown in panels D to G are representative of three independent experiments.

While differences in the interaction of the new compounds with ER/4OHTam vs the original compounds with ER/E2 are not large, they are significant. The binding cavity for the new compounds is narrower than for the original compounds; and the surface electrostatic potentials are different. The binding site for new compounds is sandwiched between the co-activator binding site and Tam binding site and is very close to the Tam molecule (Figure 4A). Thus, it is sensitive to what is bound at those sites and may affect the conformation at the co-activator binding site and AF-2 helix. Some residues on ER predicted to interact with the original compounds (Figure 4B) are different from those predicted to interact with the new compounds (Figure 4C). For example, Ile386 and Leu387 are predicted to be physically close (≤ 3 Å) to each of four active new compounds bound but not for any of four active original compounds; while Glu323 interacts with original compound A7 but not new compound 48693-K/1.

**Figure 5:**
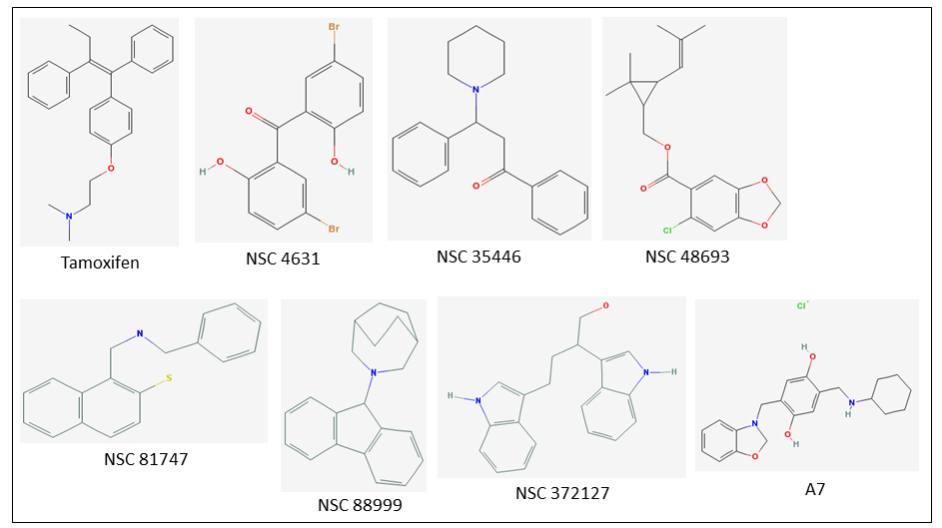
Chemical structures of six new bioactive compounds Also shown are the structures of Tamoxifen and older generation compound A7.

To test the computational models in Figure 4B-4C, we created point mutations of each residue within ER predicted to contact the compounds and compared the ability of first generation compound A7 and second generation compound 35466 to inhibit the mutant ER and wtER expressed in ER- MDA-MB-231 cells. Mutations of residues predicted to interact with 35466 conferred significant increases in IC_50_ for ER inhibition by 35446 compared to wild-type ER; while mutations of residues predicted to interact with A7 conferred large increases in the IC_50_ values for A7 compared to wtER (Table 1). Conversely, mutations of residues not predicted to interact directly with either 35446 or A7 did not alter the ability of the respective compound to inhibit ER activity. Finally, mutations of residues predicted to contact one compound but not the other significantly increased the IC_50_'s for that compound but not the other. These findings are illustrated for compound 35446 utilizing mutations of residues predicted to contact 35446 (Figure 4D-4E) and residues predicted not to contact 35446 (Figure 4E-4G). Chemical diagrams of our six new compounds, Tam, and older generation compound A7 are shown in Figure 5.

**Table 1:** Mutation analysis of estrogen receptor (ER)^1^

	A7 [^1st^ generation (old) ccompound]	NSC 35446 [2^nd^ generation (new) compound]
Mutation	IC50 (μM)	IC50 (μM)
Wild-type ER	2.5	0.8
Ile386Ala	2.5	4
Leu387Ala	2.5	5
Ile386Ala+Leu387Ala	2.5	>15
Pro325Ala	7.0	0.9
Glu353Ala	11	>10
Ile326Ala	13	4
Trp393Ala	12	>15
Arg394Ala	12	>15
Leu327Ala	12	0.9
Lys449Ala	2.0	>15
Pro324Ala	3.0	>15
Prol325Ala	7.0	0.8
Glu323Ala	13	0.9
His356Ala	11	0.8

1 ER-negative cell line MDA-MB-31 was transfected overnight with wild-type ER (wtER) or the indicated point mutant ER plus the ERE-TK-Luc reporter. Cells were treated with E2 (10 nM) and different concentrations of compound A7 or 35466-L/1 for 24-hr in phenol red-free DMEM with 5% charcoal-stripped serum (CSS). The cells were harvested for luciferase assays. From these dose-response data, IC50 values for each compound and each different ER mutation were calculated. Amino acid residues predicted to interact with compound A7 in the ER/E2 complex or with compound 35446 in the ER/Tam complex are shown in Fig. 2B and 2C, respectively.

### Inhibition of ER activity in LCC9 cells

**Figure 6:**
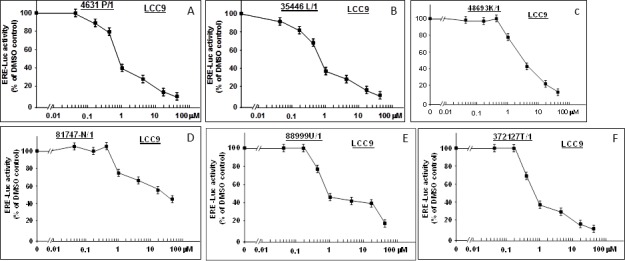
New compounds inhibit ER activity in anti-estrogen resistant LCC9 cells LCC9 cells in 24-well dishes were transfected overnight with the ERE-TK-Luc reporter plasmid (0.25 μg per well), washed, and allowed to recover for several hours in fresh culture medium. The cells were then treated with the indicated concentrations of the six bioactive compounds or vehicle only (DMSO) for 24-hr and assayed for luciferase activity. Luciferase activity is expressed as a percentage of the DMSO control. Values are means ± SEMs of four replicate wells. The data shown in each panel are representative of three independent experiments.

LCC9, which was originally derived from MCF-7, is an estrogen-insensitive cell line with a constitutively active ER that exhibits ER-dependent but E2-independent cell proliferation [15, 16]. We tested the concentration dependence of each bioactive compound for inhibition of ER activity in LCC9 (Figure 6). Four of the six compounds (4631-P/1, 35446-L/1, 88999-U/1, and 372127-T/1) showed IC_50_'s similar to those observed in MCF-7 (≤ 1 μM). The remaining compounds (48693-K/1 and 81747-N/1) yielded higher IC_50_ values (4 μM and 30 μM, respectively). Subsequent studies were carried out using 4631-P/1 and/or 35446-L/1, as representative compounds.

### 4631-P/1 disrupts the BRCA1: ER complex

**Figure 7:**
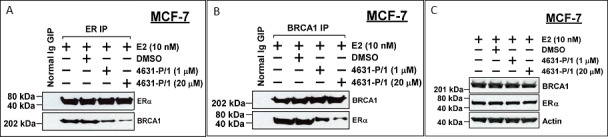
Compound 4631-P/1 disrupts the BRCA1/ER interaction in MCF-7 cells **A.**, **B.** MCF-7 cells were incubated with the indicated with compound 4631-P/1 (1 or 20 μM) or vehicle only (DMSO) plus E2 (10 nM) for 24 hr; and the cells were harvested for immunoprecipitation of ER (A) or BRCA1 (B), followed by Western blotting for ER and BRCA1. As a negative control, cells treated with vehicle (DMSO) only were subjected to an IP with normal IgG. **C.** Unprecipitated lysates from cells treated as in panel A were subjected to Western blotting to detect BRCA1, ER, or actin (loading control). The data shown in each panel are representative of three independent experiments.

Because these compounds were selected to occupy a BRCA1-binding pocket in ER, the compounds should be able to disrupt the BRCA1/ER complex. We tested the ability of 4631-P/1 to dissociate the BRCA1: ER complex by immunoprecipitation-Western blotting. At 0, 1, and 20 μM, 4631-P/1 caused concentration-dependent dissociation of ER and BRCA1; while a control IP failed to precipitate BRCA1 or ER (Figure 7A-7B). Compound 4631-P/1 had no effect on total levels of ER or BRCA1, as indicated by Western blotting of non-precipitated lysates (Figure 7C).

### 4631-P/1 and 35466-L/1 inhibit proliferation of anti-estrogen resistant cells

**Figure 8:**
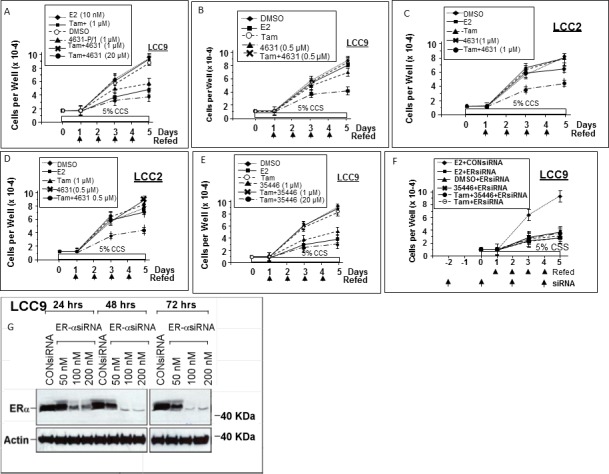
Compounds 4631-P/1 and 35466-L/1 inhibit proliferation of anti-estrogen resistant human breast cancer cells and partially restore their sensitivity to Tamoxifen LCC9 cells **A.**, **B.**, **E.**, **F.** or LCC2 cells **C.**, **D.** were seeded into 12-well dishes @ 1 × 10^4^ cells per well on day 0. Starting on day 1, cells were refed daily with fresh medium (DMEM plus 5% CSS) containing the indicated agent. In panel F, the cells were refed with medium also containing ER-siRNA or control-siRNA (100 nM) at the indicated times. Wells were counted on days 1, 3, and 5 to determine cell numbers. Values are means ± SEMs of triplicate wells. The data shown in each panel are representative of three independent experiments.

We tested two compounds for their effects on proliferation of anti-estrogen-resistant cell line LCC9 (which is E2-insensitive and resistant to Tam and Fulvestrant) and LCC2 (which is E2-independent and resistant to Tam, but not Fulvestrant). Neither E2 nor Tam significantly altered growth of LCC9 in DMEM supplemented with 5% charcoal-stripped serum (Figure 8A). However, 4631-P/1 (1 μM) inhibited LCC9 cell growth, consistent with its ability to inhibit ER activity in these cells. A combination of Tam (1 μM) and 4631-P/1 (1 μM) gave greater inhibition of cell proliferation than 4631-P/1 alone. The growth inhibitory effect of addition of Tam to 4631-P/1 was more obvious when the concentration of 4631-P/1 was reduced to 0.5 μM (Figure 8B). Similar results were observed in LCC2 (Figure 8C-8D). [Note that while LCC2 exhibits E2-independent growth, E2 can cause a slight stimulation of growth.] As for 4631-P/1, 35466-L/1 plus Tam gave greater inhibition of LCC9 proliferation than did 35466-L/1 alone (Figure 8E). Figure 8F shows that LCC9 proliferation is ER-dependent. Knockdown of ER significantly inhibited cell proliferation. In ER-siRNA-treated cells, addition of 35466-L/1 had little effect on cell proliferation, consistent with the idea that this compound acts via ER. The efficacy of the ER knockdown is shown in Figure 8G. The residual proliferation of ER-siRNA-treated LCC9 cells could be due to some ER-independent growth or incomplete ER knockdown. These data suggest that our compounds inhibit growth of Tam-resistant cells and may partially restore sensitivity to Tam.

### 4631-P/1 and 35446-L/1 disrupt interaction of ER with the ERE

**Figure 9:**
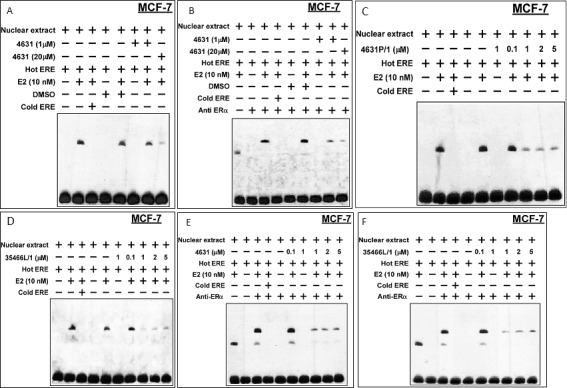
Compounds 4631-P/1 and 35446-L/1 disrupt the interaction of ER with a model ERE oligonucleotide in MCF-7 cells **A.**, **B.** Briefly, MCF-7 cells in DMEM containing 5% CSS were pre-incubated ± E2 (10 nM) and with compound 4631-P/1 (0, 1, or 20 μM) or vehicle (DMSO) for 24-hr. After the 24-hr incubation, the cells were harvested, nuclear extracts were prepared, and the extracts were reacted with labeled “hot” ERE and an excess of unlabeled “cold” ERE, as indicated, prior to electrophoresis. In panel B, the experiment was performed similarly except that an anti-ER antibody was added to generate a “supershift”. **C.**, **D.** Experiments were performed similarly to those above, except that different concentrations of compound (as indicated) were added directly to the reaction mixture containing nuclear extract and were not pre-incubated with whole cells. Panel C shows data for compound 4631-P/1, while panel D shows data for compound 35446-L/1. **E.**, **F.** These experiments were performed similarly to those in C and D, except that anti-ER antibody was added to the reaction mixture to reveal “supershifted” bands. Panel E shows data for compound 4631-P/1, while panel F shows data for compound 35446-L/1. The data shown in each panel are representative of three independent experiments.

We used electrophoretic mobility shift assays (EMSAs) of MCF-7 cells to test the effects of our compounds on binding of ER to a consensus ERE. Pre-treatment with E2 caused appearance of a band corresponding to ER bound to a labeled (“hot”) ERE oligonucleotide; and incubation with an excess of unlabeled (“cold”) ERE caused the band to disappear (Figure 9A). Pre-incubation of cells with 4631-P/1 (1 or 20 μM) caused a dose-dependent reduction of the ER/ERE complex. Addition of anti-ER antibody caused “supershift” of the ER/ERE band, confirming the presence of ER in the complex (Figure 9B). Figure 9C-9D show EMSAs in which compound was not present during pre-incubation of cells with E2 but was added directly to the nuclear extracts; and Figure 9E-9F show the corresponding supershift assays. These studies showed a greater effect when 4631-P/1 or 35446-L/(1-5 μM) was added directly to nuclear extracts, suggesting that the plasma and/or nuclear membrane may present a barrier to compound entry.

### 4631-P/1 and 35466-L/1 inhibit expression of an ER-regulated gene

**Figure 10:**
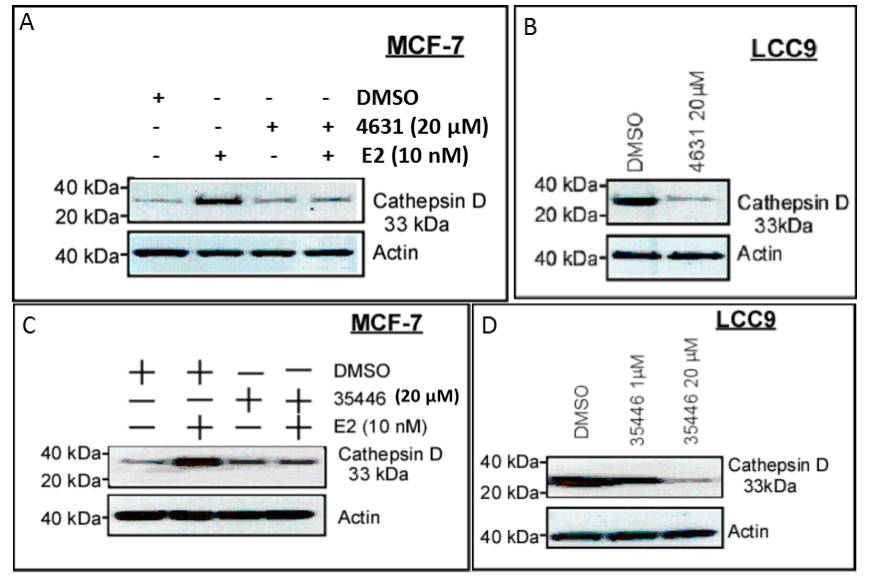
Compounds 4631-P/1 and 35466-L/1 inhibit ER-dependent expression of cathepsin D **A.**, **C.** MCF-7 cells in DMEM plus 5% CSS were subjected to the indicated treatment(s) for 24-hr and then harvested for Western blotting to detect cathepsin D or actin (control for loading and transfer). **B.**, **D.** LCC9 cells were treated with vehicle only (DMSO) or the indicated compound for 24-hr and then harvested for Western blotting for cathepsin D and actin. The data shown in each panel are representative of three independent experiments.

We used cathepsin D as a prototype E2/ER-regulated gene [17]. In MCF-7, basal cathepsin D protein levels were low, and were increased by a 24-hr exposure to E2 (10 nM) (Figure 10A, 10C). 4631-P/1 and 35466-L/1 alone had little or no effect on cathepsin D levels, but they blocked E2-stimulated expression of cathepsin D. LCC9 cells expressed cathepsin D constitutively, and the high levels of cathepsin D were decreased by a 24-hr exposure to either compound (Figure 10B, 10D).

### 4631-P/1 partially restores sensitivity of LCC9 cells to Tam-mediated inhibition of ER activity

**Figure 11:**
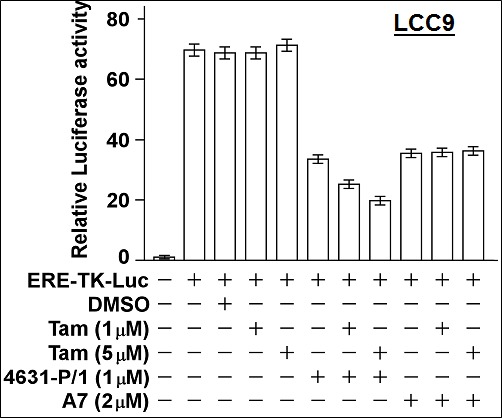
Compound 4631-P/1 partially restores the sensitivity of ER in LCC9 cells to Tamoxifen LCC9 cells in 24-well dishes were transfected overnight with the ERE-TK-Luc reporter and then incubated with the indicated agent(s) for 24-hr. The cells were then harvested for luciferase assays. Luciferase activity was expressed as a fold-change relative to the no reporter control. Values plotted are means ± SEMs of four replicate wells. The data shown are representative of three independent experiments.

Consistent with growth experiments, 4631-P/1 inhibited ER activity in LCC9 cells, while Tam alone had no effect (Figure 11). However, the combination of 4631-P/1 plus Tam gave significantly lower ER activity than 4631-P/1 alone (*P* < 0.001). In contrast, a combination of Tam plus older generation compound A7 gave no more inhibition of ER activity than A7 alone.

### Effect of compound 25446 on ER-beta (ER-β) vs ER-alpha (ER, ER-α) signaling

**Figure 12:**
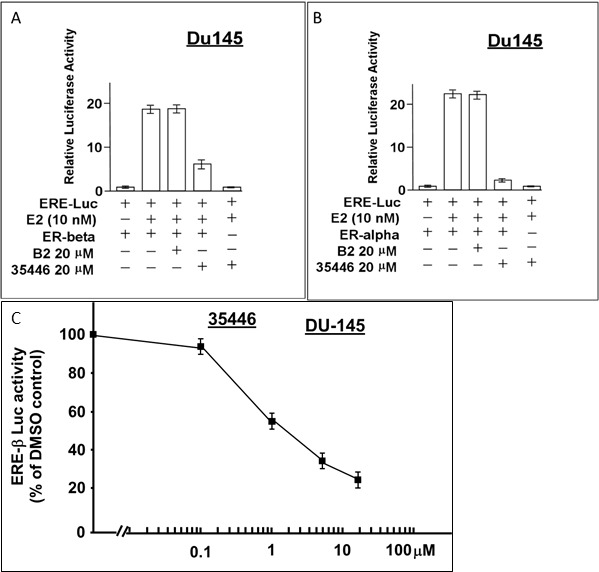
Compound 35446 inhibits the activity of ER-β Subconfluent proliferating DU-145 cells in 24-well dishes were transfected overnight with the ER=α or ER=β expression vector plus the ERE-TK-Luc reporter plasmid (0.25 μg per plasmid/well), washed, and allowed to recover for several hours in fresh culture medium. The cells were then post-incubated for 24 hr to allow gene expression, after which they were treated ± E2 (10 nM) and ± compound 35446 (20 μM) (or compound B2 as a negative control) for 24 hr and assayed for luciferase activity. Luciferase activity is expressed relative to the no E2 control. Values are means ± SEMs of four replicate wells. The data shown in each panel are representative of three independent experiments.

ER-β (also known as ESR2) is a homolog of ER-α (also known as ER or estrogen receptor 1 (ESR1)) with an overlapping but non-identical tissue distribution. Like ER-α, it is transactivated by E2 and binds to the canonical ERE; and ER-β can bind directly to ER-α and modulate its activity. Here, we compared the ability of compound 35446 to inhibit E2-stimulated ER-β activity vs ER-α activity using the ERE-luciferase reporter. Experiments were performed by transfecting ER-β or ER-α expression vectors into an ER-negative cell line (DU-145 human prostate carcinoma cells). Compound 35446 inhibited about 67% of the E2-stimulated ER-β activity but inhibited more than 90% of the ER-α activity (Figure 12A-12B). Figure 12C shows a dose response curve for inhibition of ER-β activity by compound 35446. The IC_50_ value was 2 μM, as compared with 0.8 μM for inhibition of ER-α activity (Figure 2H). Thus, the compound inhibited ER-β activity but was less efficient at inhibiting ER-β than ER-α.

### Tumor xenograft experiments

**Figure 13:**
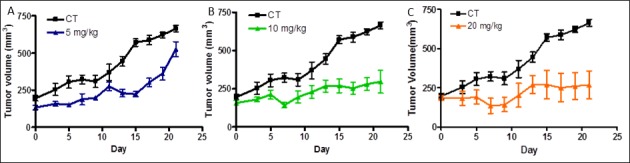
Compound NSC 35446 hydrochloride salt inhibits the growth of LCC9 breast cancer xenografts in athymic nude mice Experiments were performed as described in the Methods and Results sections. The effects of the compound administered intraperitoneally every other day at 5 mg/kg **A.**, 10 mg/kg **B.**, and 20 mg/kg **C.** are shown and compared with that of vehicle only (control, CT). Values of tumor volume are means ± SDs for N=5 mice per group.

For these studies we used LCC9 cells grown as subcutaneous tumors in Balb/c nude mice. To improve water solubility, the compound tested (NSC 35446) was prepared as the hydrochloride salt, as described in the Methods section. The mice were dosed intraperitoneally with vehicle (control) or NSC 35446 hydrochloride every other day at 5, 10, or 20 mg/kg. Treatments were initiated when the tumors reached 150-200 mm^3^. No significant changes in body weight or obvious signs of acute toxicity such as the loss of appetite, decreased activity, or lethargy were observed during the 21-day study interval. The compound appeared to be efficacious at the 10 mg/kg and 20 mg/kg doses in slowing tumor growth or causing tumor stasis (Figure 13). For most time points except the earliest, differences in size between the treated and control tumors were significant (*P* < 0.05, ANOVA, Tukey's multiple comparison test). There appeared to be some delay in tumor growth at the 5 mg/kg dose; but by day 21, the treated tumors were approaching the control tumors in size. The T/C ratios (*ie*., ratios of the changes in volume of treated tumors to that of control tumors) on day-21 were: 0.77 (5 mg/kg), 0.24 (10 mg/kg), and 0.15 (20 mg/kg). These findings suggest dose-dependent inhibition of LCC9 tumor growth by NSC 35446 hydrochloride.

## DISCUSSION

We described and partially characterized a novel group of small molecule compounds that act as ER antagonists by binding to a putative BRCA1-binding cavity that is distinct from the ligand-binding pocket (LBP) and the coactivator-binding pocket in ER. Development of small molecule compounds that inhibit or mimic the binding interaction of two proteins is problematic because protein:protein interactions often occur over broad smooth surfaces that are not amenable to to small molecules. In a high resolution study of the BRCA1: ER interaction, we proposed a three-dimensional model of a partial BRCA1: ER complex in which the interaction occurred over a broad area [18]. However, we were able to identify a potential BRCA1-binding cavity that is narrow enough and deep enough to accommodate a small molecule with a relatively tight fit. Evidence that these compounds inhibit ER activity in a manner related to BRCA1 comes from the finding that one compound (4631-P/1) disrupted the BRCA1:ER association in cultured cells.

Two classes of ER antagonists, SERMs (selective ER modulators) and SERDs (selective ER degraders) are currently used for treatment of ER+ breast cancer and/or in breast cancer prevention; and additional SERMs are currently under development [19-21]. SERMs currently in use include Tam, Toremifene, Clomifene and Raloxifene. All SERMs as well as Fulvestrant (a SERD) bind to the LBP of ER. Since our compounds do not bind to the LBP, their mechanism of action differs from SERMs and SERDs. Evidence that our compounds work differently comes from their ability to inhibit ER activity and cell proliferation in LCC9, a cell line that is resistant to Tam and Fulvestrant and LCC2, a cell line resistant to Tam, but not Fulvestrant [15, 16]. A modified form of compound 35446 (hydrochloride salt) inhibited the growth of LCC9 tumor xenografts at doses (10 and 20 mg/kg every other day) that yielded no acute toxicity, suggesting the compound can be administered safely and effectively.

Many different mechanisms for anti-estrogen resistance have been identified [1, 22]. Some of these (*eg*., loss of ER expression due to promoter hypermethylation or other mechanisms) are not amenable to treatment with any ER antagonist. Sensitivity of anti-estrogen-resistant breast cancer cells to our compounds requires that tumor cells retain ER protein and remain dependent upon ER for proliferation and/or survival. As we have tested these compounds in a small number of anti-estrogen-resistant cell types, it is unclear if the compounds will inhibit all ER+ tumor cells that are resistant to conventional anti-estrogens or only a subset of these tumor cells.

So far, we have not observed any ER agonist activity for these compounds. SERMs like Tam can have both agonist or antagonist activity in different contexts and in different tissues and organs. Thus, while Tam inhibits E2-stimulated ER activity in MCF-7 cells, it stimulates ER activity in MCF-7 cells in the absence of E2 [10, 13]. Tam agonist activity can be beneficial (*eg*., in bone, where Tam acts like E2 to increase bone density) or problematic (*eg*., in the uterus, where long-term use can cause uterine hyperplasia and cancer) [23-25]. It remains to be determined how our compounds affect ER activity in the other ER+ tissues. Despite their general similarity, different SERMs have different tissue-specific activity profiles. For example, Tam and Raloxifene inhibit ER activity in the breast but Raloxifene, unlike Tamoxifen, does not act as an agonist in the uterus. Both agents act as agonists in bone, and both have similar effects in the cardiovascular system, where they increase the risk of thromboembolism [26, 27].

It is not surprising that our compounds inhibit ER activity in Tam-resistant cells because they differ from Tam in mode of interaction with ER. But it was surprising that these compounds partially restore Tam sensitivity in LCC9 and LCC2 cells. This is consistent with a model in which binding of a compound to the ER/Tam complex cells alters the conformation of ER so that it is re-sensitized to Tam. Alternatively, if the ER in LCC9 cells is configured so that it does not bind Tam, ligation to a compound might alter the ER conformation so that it can bind Tam.

A somewhat surprising finding was the observation that our compounds disrupted the interaction between ER and an ERE oligonucleotide in EMSAs and supershift assays using MCF-7 nuclear lysates. Similar results were obtained for LCC9 anti-estrogen resistant cells (data not shown). These findings suggest that our compounds may stabilize ER in a conformation that is unsuitable for binding to DNA. However, it is cautioned that the effect of the compounds on the ER: ERE interaction may not be direct, since these experiments were carried out using nuclear lysates and not under cell-free conditions. These findings suggest that our compounds, which were not designed to target the DNA binding domain of ER may cause a conformational change that results in the disruption of the ER: ERE interaction, just as E2 can cause a profound conformation change in ER by binding to the ligand-binding pocket (LBP).

Our ER mutation analyses indicate that original (first) and new (second) generation compounds exhibit distinct patterns of inhibition of ER activity. Thus, mutation of any of the residues unique for the BRCA1 mimetic pocket of first generation compounds blocked ER inhibitory activity of first generation but not second generation compounds; and conversely, mutation of any of the residues unique for the BRCA1 mimetic pocket of second generation compounds block the ER inhibitory activity of second generation but not first generation compounds. The differences in structure between the two pockets may be the related to why only the second generation compounds synergize with Tam in Tam-resistant LCC9 and LCC2 cells, but exactly how and why this is the case must await detailed structural biologic studies. In this regard, I386 and L387 may be important for the synergy with Tam of second generation compounds (see Figure 1C).

Caboni and Lloyd [28] have reviewed the targeting of sites in nuclear receptors other than the LBP. Most of the small molecule inhibitors that target non-LBP sites in both ER and other nuclear receptors in that review target the coactivator binding site, although some targeted the DNA-binding domain or other sites Most also work at significantly higher concentrations than do our compounds (IC_50_ about 0.8 μM for our compounds). Our binding site is distinct from the LBP and the coactivator binding site, but it is close enough to either site that it may allosterically influence these sites.

Both ER knockdown with RNAi [35] and the ER antagonists induce growth inhibition, implying that some functions within LCC9 cells remain dependent upon constitutive ER action. Other components of the cell fate machinery in these cells are likely to act independent of ER function. The complexity of signaling in endocrine resistance has been reviewed extensively and likely includes both ER-dependent and ER-independent signaling to regulate the balance between prodeath apoptosis and prosurvival autophagy [36].

Finally, we tested the ability of compound 35446 to inhibit ER-β activity and found that it significantly inhibited ER-β activity when ER-β was transfected into ER- cells; but the inhibition was less than that of ER-α transfected into the same cell type. These findings are consistent with the high degree of homology between the ligand binding domains of ER-β and ER-α, as well as the near identity of the DNA-binding domain of these two receptors.

## MATERIALS AND METHODS

### Cell lines and culture

MCF-7, LCC9, LCC2, DU-145, and MDA-MB-231 cells were obtained from the Lombardi Comprehensive Cancer Center Tissue Culture Shared Resource and cultured as described earlier [13, 15, 16, 18, 29].

### Reagents

17β-estradiol (E2), and Tam, and 4OH-Tam were obtained from Sigma (St. Louis, MO), and R5020 was purchased from PerkinElmer Life Sciences (Waltham, MA) These agents were dissolved in DMSO (Sigma) and added to culture medium at the time of assays. MTT dye ((3-(4,5-dimethylthiazol-2-yl)-2,5-diphenyltetrazolium bromide) was obtained from Sigma. The compounds screened in bioassays were obtained from the NCI/DTP Open Chemical Repository through the DTP website: http://dtp.cancer.gov in powder form.

### Generation of NSC 35446 hydrochloride salt

A solution of free base 1,3-diphenyl-3-(piperidin-1-yl)propan-1-one (NSC 35446) (2 g, 6.8 mmol) in anhydrous diethyl ether (100 ml) was placed to stir on iced-water bath. After complete compound dissolution, a solution of HCl (concentrated) in ether (1:9 v/v) was added in a dropwise manner until complete precipitation of the salt (pH 4). The solid was separated by filtration and washed with diethyl ether (3 × 25 ml) to remove excess acid. The product was recrystallized from ethanol and air-dried at 25°C to yield 1.2 g (53%) of compound as a white crystalline salt.

### Expression vectors and reporters

Wild-type BRCA1 expression vector was created by cloning full-length BRCA1 cDNA into the pcDNA3 vector (Invitrogen, Carlsbad, CA) [30]. The reporter ERE-TK-Luc is composed of the vitellogenin A2 enhancer (ERE) controlling a minimal thymidine kinase promoter (TK81) and luciferase in plasmid pGL2 [31]. The progesterone-responsive reporter MMTV-Luc was described earlier [32]. The mutant ER expression vectors were created by site directed mutagenesis of ER cDNA in the pCMV-ER vector.

### siRNAs

siRNAs used herein were as follows: ER-siRNA: Sense 5′-CAGGCACAUGAGUA ACAAATT-3′ and antisense 5′-UUUGUUACUCAUGUGCCUGAT-3′ (Ambion/Life Technol-ogies, Washington, DC); and negative control-siRNA (AM4461, Ambion). Subconfluent proliferating cells were treated with ER- or control-siRNA (100 nM) using siPORT Amine transfection reagent (Ambion). Western blotting revealed that a minimum of 48-hr exposure to 100 nM of ER-siRNA was required to obtain >75% reduction of ER.

### Assays of ER activity

Subconfluent cells in 24-well dishes were transfected overnight with 0.25 μg of each indicated vector plus the ERE-TK-Luc reporter in serum-free DMEM containing Lipofectamine2000 (Life Technologies, Washington, DC). Total transfected DNA was kept constant by addition of control vector. The cells were washed, incubated in phenol red-free DMEM containing 5% charcoal-stripped serum (obtained from the Tissue Culture Shared Resource) (0.2 ml/well) ± E2 (10 nM) ± the indicated compound for 24-hr, and harvested for luciferase assays. For each assay condition was tested in quadrupicate. To monitor the transfection efficiency, cultures were co-transfected with plasmid pRSV-β-gal (Promega, Madison, WI) to visualize transfected cells by X-gal staining.

To determine the effect of compound on ER-β activity, an ER- β expression plasmid (cat. no.: 35562, Addgene, Cambridge) was co-transfected into ER- DU-145 human prostate carcinoma cells along with the ERE-TK-Luc reporter plasmid (0.25 μg of each plasmid) and the cells were incubated overnight to allow gene expression. The cells were then treated ± E2 (10 nM) and ± the indicated compound for 24-hr, and harvested for luciferase assays as above.

### Assay of progesterone receptor (PR) transcriptional activity

PR assays were carried out in T47D cells using the synthetic progestin R5020 (10 nM) to activate PR and the MMTV-Luc reporter as a readout for PR activity, as described before [32].

### MTT assays of cell viability

Assays were performed as described before [30]. After the indicated treatment, cells in 96-well dishes were solubilized and assayed for MTT dye reduction. Cell viability was expressed as the amount of dye reduction relative to untreated control cells. and calculated as means ± SEMs for 10 replicate wells.

### Cell proliferation assays

Assays were performed using as the growth medium phenol red-free DMEM containing 5% charcoal-stripped serum. Briefly, proliferating cells were harvested using trypsin, counted, inoculated into 12-well dishes at 1 × 10^4^ cells per well on day 0 and allowed to attach and recover for 24 hr. The cells were then treated with the indicated agents for up to five days, with daily refeeding with fresh medium and agents. Triplicate wells were counted by Coulter Counter on days 1, 3, and 5. For experiments using siRNAs, cells were treated with the indicated siRNA (100 nM) starting on day -2, and fresh siRNA was added on days 0, 2, and 4.

### EMSA and supershift assays

Double-stranded oligonucleotides containing a consensus ERE were obtained from Santa Cruz Biotechnology (sc-2858). Sequences of the oligonucleotides were: sense 5′-GGATCTAGGTCACTGTGACCCCGGATC-3′ and antisense: 3′-CCTAGATC CAGTGACACTGGGGCCTAG-5′. Oligonucleotides were 3′-end-labeled using a Biotin Kit (Thermo Scientific, Rockford, IL). DNA binding reactions were carried out using nuclear extracts of MCF-7 cells that were treated ± E2 (10 nM) and ± the indicated compound for 24-hr. Treated cells were harvested and nuclear extracts were prepared using the NE-PER Nuclear and Cytoplasmic Extraction Reagents (Thermo Scientific). Aliquots of nuclear extract protein (2 μg) were incubated with gel-shift binding buffer (LightShift Chemiluminescent EMSA Kit, Thermo Scientific) for 30 min at 25°C. After incubation, 40 fmol of biotin-labeled ERE-containing oligonucleotides were added, and the mixture was re-incubated for 30 min at 25°C. Reaction products were loaded electrophoresed on a 6% DNA retardation gel (Invitrogen) and transferred onto a nylon membrane. Biotin-labeled DNA was detected using the Chemiluminescent Nucleic Acid Detection Module (Thermo Scientific). To test the specificity of binding, a 40-fold excess of unlabeled (“cold”) oligonucleotide was added to one of the reactions along with the biotin-labeled (“hot”) oligonucleotide. *Note*: In some experiments, the compound was added to the reaction mixture after the nuclear extract was prepared. Supershift assays were performed as above, except that before addition of the hot ERE oligonucleotide, nuclear extracts were incubated with 2-μg of anti-ER IgG (rabbit polyclonal, HC-20, Santa Cruz) for 30 min at 25°C.

### Immunoprecipitation (IP)

After the indicated treatments, cells were harvested, and whole cell extracts were prepared in IP buffer [10 mM Tris-HCl (pH 7.4), 150 mM NaCl, 1 mM EDTA, 1 mM EGTA, 1% Triton X-100, 0.5% IGEPAL CA-630 (Sigma), 10% glycerol, 1 mM sodium orthovanadate, and protease inhibitor cocktail (Santa Cruz)] [11, 18]. Each IP was carried out using 2-μg antibody and 500-μg extract protein. The extracts were incubated anti-ER antibody H184 (sc-7207, rabbit polyclonal IgG, Santa Cruz) or with a combination of anti-BRCA1 mouse monoclonals (Ab-1+Ab-2+Ab-3, Calbiochem, San Diego, CA); and the precipitated proteins were collected using protein A/G agarose (Santa Cruz). After low-speed centrifugation to remove the supernatant, the agarose was washed with PBS, collected in boiling sample buffer (Santa Cruz), and subjected to SDS-PAGE and Western blotting. For each experiment, a control IP using an equal quantity of normal mouse or rabbit IgG (Santa Cruz) was carried out.

### Western blotting

After the indicated cell treatment(s), the cells were harvested, and whole-cell lysates were prepared using RIPA buffer (Santa Cruz) [11, 18]. Equal aliquots of whole-cell protein (either 100 μg unprecipitated whole-cell lysate or one half of the precipitated protein from 500-μg whole-cell lysate) were electrophoresed on 4-12% SDS-polyacrylamide gradient gels, transferred to nitrocellulose membranes (Millipore, Bedford, MA), and blotted using primary antibodies against BRCA1 (C-20, rabbit polyclonal, 1:200 dilution; Santa Cruz), cathepsin D (R-20, goat polyclonal, 1:300; Santa Cruz), ER (F10, mouse monoclonal, 1:500; Santa Cruz), or actin (goat polyclonal, sc-1615, 1:400; Santa Cruz). The membranes were blotted with the appropriate secondary antibodies (1:1000; Santa Cruz), and the blotted proteins were visualized using an electrochemiluminescence detection system (Amersham Biosciences), with colored markers (Bio-Rad Laboratories, Hercules, CA) as molecular size standards.

### *In silico* screening of small molecules

Similarly to the previous screening strategy to identify “BRCA1-mimetic” compounds based on ER bound with E2 [13], an *in silico* screening with AutoDock (ver. 4, The Scripps Research Institute; [33]) and AutoDockTools (ADT) was set up based on the model structure of the BRCA1-binding interface of the ER LBD in complex with OHT (4-hydroxytamoxifen) (PDB: 2ERT) [34]. The BRCA1 binding pocket is equivalent to the one on ER in complex with E2, but the new site is redefined for ER in complex with OHT. The virtual screening library was the NCI/DTP “Diversity Set” (about 1,990 compounds), selected from over 140,000 compounds available for distribution from the DTP repository (http://dtp.nci.nih.gov/branches/dscb/repo_open.html). The selection of the diversity set is based on diversity of pharmacophores, chemical structure, pharmacologically desirable features, and availability and purity of the compounds. In general, the default parameters were used for AutoDock docking and the docking results were ranked based on their binding energies, clustering of their 10 lowest energy binding conformations, and interactions with ER, such as hydrogen bonds and hydrophobic interactions with visual inspections.

### Animal experiments

Athymic Balb/c nude mice were purchased from Charles River. Mice were housed 5 per cage with microisolater tops and provided food (Furina mice chow) and water ad libitum. The light cycle was regulated automatically (12 hours light/dark cycle) and temperature 23±1°C. Animals were allowed to acclimate to this environment for one week prior to usage. The Georgetown University Animal Care and Use Committee approved all animal studies in accordance with NIH guidelines. The stock solution of NSC 35446 hydrochloride was dissolved in DMSO at 200 mg/ml. The working solution was prepared using 10% Kolliphor ELP (Sigma-Aldrich), 3% PEG400 (Hampton Research, HR2-603); and 87% PBS to give a 1 mg/ml solution. Female Balb/c nude mice (18-22g) were injected with LCC9 cells (3 × 10^6^ cells in a volume of 0.3 ml) in the subcutaneous tissue of the right axillary region. The mice were randomly sorted into groups with N=5 mice per group; and treatments were initiated when the tumors reached 150-200 mm^3^. The tumor-bearing mice were given intraperitoneal injections of vehicle only (control), 5 mg/kg, 10 mg/kg, or 20 mg/kg of NSC 35446 hydrochloride every other day. The tumor size of each mouse was measured by caliper and calculated by the formula: length × width × width/2 and the body weight was recorded.

### Statistical methods

Where appropriate, statistical comparisons were made using the two-tailed Student's t test or by ANOVA.
